# A Highly Sensitive Cell-Based TLR Reporter Platform for the Specific Detection of Bacterial TLR Ligands

**DOI:** 10.3389/fimmu.2021.817604

**Published:** 2022-01-11

**Authors:** Katharina Radakovics, Claire Battin, Judith Leitner, Sabine Geiselhart, Wolfgang Paster, Johannes Stöckl, Karin Hoffmann-Sommergruber, Peter Steinberger

**Affiliations:** ^1^ Division of Immune Receptors and T Cell Activation, Institute of Immunology, Center for Pathophysiology, Infectiology and Immunology, Medical University of Vienna, Vienna, Austria; ^2^ Department of Pathophysiology and Allergy Research, Center for Pathophysiology, Infectiology and Immunology, Medical University of Vienna, Vienna, Austria; ^3^ Clinical Cell Biology and FACS Core Unit, St. Anna Children´s Cancer Research Institute (CCRI), Vienna, Austria; ^4^ Division Regulation of the Immune System, Institute of Immunology, Center for Pathophysiology, Infectiology and Immunology, Medical University of Vienna, Vienna, Austria

**Keywords:** toll-like receptor, TLR, biosensor, bacterial contamination, reporter cell

## Abstract

Toll-like receptors (TLRs) are primary pattern recognition receptors (PRRs), which recognize conserved microbial components. They play important roles in innate immunity but also in the initiation of adaptive immune responses. Impurities containing TLR ligands are a frequent problem in research but also for the production of therapeutics since TLR ligands can exert strong immunomodulatory properties even in minute amounts. Consequently, there is a need for sensitive tools to detect TLR ligands with high sensitivity and specificity. Here we describe the development of a platform based on a highly sensitive NF-κB::eGFP reporter Jurkat JE6-1 T cell line for the detection of TLR ligands. Ectopic expression of TLRs and their coreceptors and CRISPR/Cas9-mediated deletion of endogenously expressed TLRs was deployed to generate reporter cell lines selectively expressing functional human TLR2/1, TLR2/6, TLR4 or TLR5 complexes. Using well-defined agonists for the respective TLR complexes we could demonstrate high specificity and sensitivity of the individual reporter lines. The limit of detection for LPS was below 1 pg/mL and ligands for TLR2/1 (Pam3CSK4), TLR2/6 (Fsl-1) and TLR5 (flagellin) were detected at concentrations as low as 1.0 ng/mL, 0.2 ng/mL and 10 pg/mL, respectively. We showed that the JE6-1 TLR reporter cells have the utility to characterize different commercially available TLR ligands as well as more complex samples like bacterially expressed proteins or allergen extracts. Impurities in preparations of microbial compounds as well as the lack of specificity of detection systems can lead to erroneous results and currently there is no consensus regarding the involvement of TLRs in the recognition of several molecules with proposed immunostimulatory functions. This reporter system represents a highly suitable tool for the definition of structural requirements for agonists of distinct TLR complexes.

## Introduction

Toll-like receptors (TLRs) have been widely studied since the discovery of *Drosophila* Toll in 1985 (1) and the description of its involvement in *Drosophila* antifungal immunity in 1996 (2). In humans, four well-characterized TLR complexes can be expressed on the cell surface: TLR2 as a heterodimer with TLR1 or TLR6, TLR4, which forms a complex with MD-2 and CD14, and TLR5, which forms homodimers. Additionally, TLR3, TLR7, TLR8 and TLR9 are expressed in intracellular compartments. TLRs are present on a variety of human cells, including cells of the innate and adaptive immune system, and they play an important role in the initiation of immune responses. Although PRRs have initially been described as receptors of the innate immune system, an important role for TLRs in the adaptive immunity has been revealed in recent years (3). For example, TLR signals mediate the activation of antigen presenting cells like dendritic cells and B cells, thereby dramatically augmenting their capacity to induce T cell responses (4–6).

Consequently, TLRs play an important role in health and disease. Their activation is crucial for an efficient immune response against pathogens, but TLR signaling can also be detrimental for human health, for example in sepsis. Genome-wide association studies have found a link between single nuclear polymorphisms in TLRs and allergic sensitization and both pro- and anti-oncogenic effects have been attributed to TLR activation on tumour cells and tumour infiltrating lymphocytes (7–12). Due to their immunostimulatory capacity, TLR ligands are an emerging tool in vaccinology. Monophosphoryl lipid A (MPLA), a derivative of lipopolysaccharide (LPS) and potent agonist of TLR4, is already used in vaccines, while the TLR5 ligand flagellin is currently investigated as potential adjuvant (13–16).

Contamination with TLR ligands is a major concern for the production of proteins for research but also for protein-based therapeutics. Especially LPS, a ligand of the TLR4/MD-2/CD14 complex, and the TLR5 ligand flagellin frequently contaminate protein preparations if bacterial expression systems like *E. coli* are used (17, 18). Moreover, several human proteins and lipopeptides, as well as the widely used reagent bovine serum albumin, bind to LPS, and removing it during the purification process remains a challenge (19, 20). Co-purified TLR ligands have led to confounded results for immunostimulatory proteins supposedly directly triggering TLRs. Prominent examples are heat shock proteins or saturated fatty acids (21–27). Although contaminations with LPS are the main concern, other TLR ligands also influence immune responses (28–32). Therefore, their presence might render therapeutic proteins immunogenic or peril the outcome of experiments with recombinant proteins (33). While LPS can be detected by the limulus amebocyte lysate (LAL) assay or equivalent assays based on recombinant Factor C, which are routinely used for the analysis of biological and pharmaceutical products, most bacterial ligands of TLRs other than TLR4 are not detected by those assays. As a consequence, screening for the presence of such TLR ligands is frequently not performed for therapeutic proteins (34). Accordingly, suppliers of recombinant proteins for research frequently provide information on endotoxin content (usually measured by LAL test or equivalent assays), but tests to detect the presence of other TLR ligands are usually not performed. This is also true for complex protein preparations such as allergen extracts used in research but also for the diagnosis and treatment of allergies. Pelst et al. proposed that TLR ligands might interfere with induction of allergen tolerance by sublingual immunotherapy (35). Therefore, there is great interest in tools for the detection of LPS, but also of other TLR ligands. Electrochemical detection systems exploiting TLRs immobilized on chips, as well as TLR5-containing liposomes for the detection of flagellin by surface plasmon resonance have been developed (36–38). However, their readout relies on instruments not readily available in many laboratories. Cell-based systems that express human TLRs are robust and cost-effective platforms for the detection of TLR ligands. However, in many cases such systems are based on innate immune cells such as primary monocytes or dendritic cells or myeloid cell lines such as THP-1 or RAW264.7. Innate immune cells express several TLRs and, consequently, they cannot be used to measure ligands for specific TLR complexes. Human embryonic kidney (HEK) cell-based reporter cells expressing TLRs of choice are frequently used for the detection of TLR ligands of interest, but their specificity is hampered by the expression of several endogenous TLRs and other PRRs.

Here we describe the use of transcriptional NF-κB::eGFP reporter cells based on the human T cell line Jurkat JE6-1 to generate a highly sensitive platform for the detection of ligands for cell surface resident human TLRs. We demonstrate the utility of this JE6-1 TLR reporter platform to specifically detect ligands for TLR2/1, TLR2/6, TLR4 and TLR5 and to identify agonists for different TLR complexes in biological samples.

## Methods & Materials

### Cell Culture and Reagents

JE6-1 reporter cells (39, 40), THP-1 reporter cells (41), and HEK-Blue™-hTLR4 (InvivoGen, San Diego, CA) cells were maintained in RPMI 1640 supplemented with 100 μg/mL streptomycin, 100 U/mL penicillin and 10% heat-inactivated fetal calf serum (all Sigma Aldrich, St. Louis, MO) at 37°C in a humidified atmosphere with 5% CO_2_. The TLR agonists Pam3CSK4, Fsl-1, LPS-B5 std. and ultrapure, Fla-ST std., ultrapure and recombinant, Fla-BS, Poly(I:C), Resiquimod (R848), Imiquimod (R837), CpG (ODN 2006), PGN-BS, -EK, -SA and LTA-SA (LTA-SA purified) were purchased from *InvivoGen*. LPS-B8 (from *E. coli* 0127:B8), Diprovocim-1, Phorbol-12-myristat-13-acetat (PMA) and ionomycin were obtained from Sigma Aldrich. A list of the TLR ligands used is provided in **Table S1**. Anti-human CD282 (TLR2, clone QA16A01) antibody coupled to Alexa Fluor^®^ 647 was purchased from BD Biosciences (San Jose, CA).

Allergen extracts were produced in house as described before (42, 43) from pollen derived from birch tree (*Betula pendula*), timothy grass (*Phleum pratense*), mugwort (*Artemisia vulgaris*), ragweed (*Ambrosia artemisiifolia*) as well as house dust mites [*Dermatophagoides pteronyssinus*, HDM(2)], which were obtained from Allergon (Thermo Fisher Scientific, SE). HDM(1) extract was prepared from 0.3 mg *Dermatophagoides farinae* (Allergon, Thermo Fisher Scientific, Batch 495518303) with 5 ml of extraction buffer (PBS pH 7.2, containing protease inhibitor). The mixture was sonicated for 10 minutes to achieve good solubility and gently stirred overnight at 4°C. The next day, after 30 minutes of centrifugation (21000 g, 4°C) the protein concentration of the supernatant was determined and aliquots were stored at -20°C. HDM(3) (Item Number XPB70D3A2.5, Lot 290903) and HDM(4) (Item Number XPB91D3A2.5, Lot 381018) were purchased from Stallergenes Greer (Lenoir, NC).

### CRISPR/Cas9

The TLR5 reporter cells stably expressing an NF-κB::eGFP reporter gene have been previously established in our laboratory (39, 40). The TLR5 gene was knocked out by CRISPR/Cas9 using an Amaxa Nucleofector II (Lonza, Basel, CH) for ribonucleoprotein electroporation. 1.5 x 10^6^ TLR5 reporter cells were mixed with pre-incubated ribonucleoprotein consisting of 30 pmol Cas9 protein and 150 pmol sgRNA targeting the sequence 5´-ATGAGCTCGAGCCCCTACAA-3´ (both Integrated DNA Technologies, Coralville, IA). Electroporation was performed in Chica buffer (44) with final 10 μM Electroporation Enhancer (Integrated DNA Technologies), using the electroporation program X-001. Cells without reactivity towards flagellin (no eGFP expression after 24 h incubation with 200 ng/mL Fla-ST std.) were sorted using a Sony SH800 cell sorter (Sony Biotechnology, San Jose, CA) with targeted sorting into a 96-well plate to obtain single cell clones. TLR5 knockout was confirmed by PCR amplification of the target region using the primer pair TLR5-forward (5´-GCTCCTTTGATGGCCGAATA-3´) and TLR5-reverse (5´-CCAGGCCAGCAAATGTGTTC-3´) (Sigma Aldrich). The forward primer was used for sequencing.

The TLR6 gene in the TLR2/1/6 (to obtain TLR2/1) and TLR2/6 (to obtain TLR2-only) reporter cells was knocked out using the Neon™ Transfection System 10 µL Kit (ThermoFisher Scientific, Waltham, MA). Pre-designed TLR6.AB crRNA targeting the sequence 5´- ATTCAGTAAGGTTGAACCT-3´ in the TLR6 gene was complexed with tracrRNA in a 1:1 molar ratio to generate gRNA (Integrated DNA Technologies). 5 x 10^5^ cells were electroporated with ribonucleoproteins consisting of 22 pmol gRNA and 18.6 pmol Cas9 protein with final 2.2 μM Electroporation Enhancer (Integrated DNA Technologies) in Buffer R (ThermoFisher Scientific). Cells were electroporated with 1600 V, for 10 ms, three times. Single cell clones were generated by limiting dilution culturing. Knockout of the TLR6 gene was verified by amplifying the region of interest with the primers TLR6-forward (5´-CAAGTTCAACCAGGATTTAGAATATTTGGATTTATC-5´) and TLR6-reverse (5´- AGAAATCAGCCGATGGGTGG-3´) (Sigma Aldrich). The forward primer was used for sequencing.

### Introduction of TLR2, TLR1 and TLR4/MD-2/CD14 Into JE6-1 Reporter Cells

Retroviral expression constructs coding for TLR4, MD-2 and CD14 were described before (41). TLR4, MD-2 and CD14 were simultaneously transduced into TLR non-responder cells. Positive eGFP-expressing cells were sorted after stimulation with LPS (300 ng/mL) for 24 h using a Sony SH800 cell sorter. Single cell clones were established by limiting dilution culturing.

Sequences encoding human TLR2 and TLR1 were cloned into the retroviral expression vector pCJK2 (45). The TLR2 construct was expressed in TLR non-responder cells and TLR2-expressing cells were sorted on a Sony SH800 cell sorter using an AlexaFluor647-coupled anti-human CD282 (TLR2) antibody (BD Biosciences). Single cell clones were established and a clone with high reactivity towards Fsl-1 (TLR2/6 ligand) was chosen as TLR2/6 reporter cell line. Into the TLR2-positive sorted cell pool, TLR1 was transduced to establish TLR2/1/6 reporter cells. Cells were sorted for reactivity against Pam3CSK4 (TLR2/1 ligand) by sorting eGFP-expressing cells after activation with 100 nM Pam3CSK4 for 24 h on a Sony SH800 cell sorter. Single cell clones were established and a clone with high reactivity towards Fsl-1 (TLR2/6 ligand) and Pam3CSK4 (TLR2/1 ligand) was chosen as TLR2/1/6 reporter cell line.

### JE6-1 and THP-1 Reporter Cell Stimulation

5 x 10^4^ reporter cells were cultivated with the indicated stimulus in duplicates in a final volume of 100 μl in 96-well plates for 14 – 24 h. Cells were harvested and eGFP expression was measured by flow cytometry. Flow cytometry data were acquired at a FACS Calibur with CellQuest software and an LSRFortessa (both BD Bioscience). Data were analysed using FlowJo software vesion 10.6.1 (Tree Star, Ashland, OR). Fold induction of eGFP expression was calculated for each replicate of a given reporter cell in a given experiment individually by dividing the gMFI by the average gMFI of that reporter cell at unstimulated conditions in the same experiment.

### Bacterial Protein Production

The human complement split product C4dg was produced in *E. coli* BL21 and ClearColi™ BL21 as described before (41).

### LPS Detection by HEK-Blue™-hTLR4 Reporter Cells and Recombinant Factor C Assay

HEK-Blue-hTLR4 cells carrying an embryonic alkaline phosphatase (SEAP) reporter construct were a kind gift by Johannes Stöckl (originally InvivoGen). Cells were grown in DMEM (glutamine, sodium pyruvate and sodium bicarbonate) supplemented with 10% heat-inactivated fetal calf serum at 37°C and 5% CO_2_. Cells were seeded at a density of 2.5 x 10^5^/mL in a 96-well flat-bottom plate and LPS-B5 ultrapure (*InvivoGen*) was added to the cells. After 16 h, supernatants were collected and SEAP activity (NF-κB activation) was assessed using detection medium QUANTI-Blue, prepared according to manufacturer recommendations (Invivogen).

The recombinant Factor C-based assay Endozyme II was purchased from Hyglos (Bernried, DE). All samples and solutions were brought to room temperature before use. The supplied control standard endotoxin (LPS-O55:B5) as well as test samples were reconstituted in endotoxin-free water (supplied) and dilutions were prepared. 100 μL of standard solutions, samples and controls were transferred to a black, flat-bottom, 96-well microplate, respectively. Subsequently, 100 μL assay reagent, consisting of previously mixed assay buffer, substrate and recombinant Factor C enzyme in a ratio of 8:1:1 were added. The plate was then incubated at 37°C for 60 minutes and the fluorescence intensity was measured with excitation at 380 nm and emission at 448 nm in relative fluorescence units (RFU). Fold change was calculated by dividing the RFU of a given sample by the mean RFU of the negative control in the same experiment.

### Statistics

Statistical analysis was performed using GraphPad Prism version 7 (GraphPad Software, Inc., La Jolla, CA). Curve fitting in titration curves was performed using the equation [Agonist] vs. response – variable slope (four parameters) with the fitting method “Least squares (ordinary) fit”. x-values at y = 1.5 were interpolated from the standard curve with a 95% confidence interval. To compare TLR4 and TLR5 reporter cell activation by different flagellin preparations, two-way ANOVA was performed. Within each row, columns were compared. Each cell mean was compared to the control cell mean (Fla-ST std.). In aligned dot plot, individual values are shown with a line at the mean. Titration curves show the mean and error (standard deviation). Scatter dot plots show each replicate, the mean and standard deviation.

## Results

### Jurkat Reporter Cells Are Activated by TLR5 Ligands

**Figure 1:**
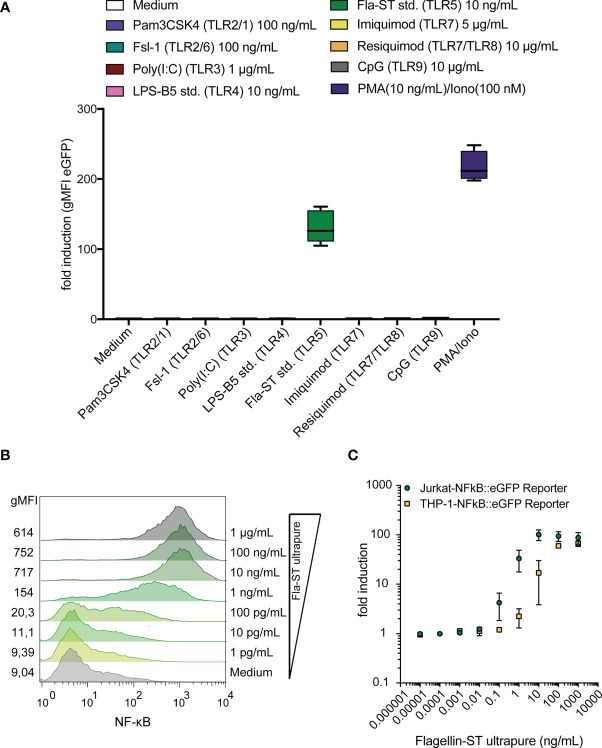
Jurkat-NF-κB::eGFP reporter cells detect the TLR5 ligand flagellin with high sensitivity and specificity. **(A)** eGFP expression in Jurkat JE6-1 cells stably transfected with an NF-κB::eGFP reporter gene treated with different TLR ligands and PMA/Ionomycin as a positive control for 24 h (n = 3 independent experiments performed in duplicates). **(B)** Representative histograms of eGFP expression in JE6-1 reporter cells stimulated with flagellin at the indicated concentrations (experiment shown is representative for three experiments independently performed). **(C)** Concentration-response curves upon stimulation of Jurkat-NF-κB::eGFP and THP-1-NF-κB::eGFP reporter cells with flagellin are shown (n = 3 for Jurkat and n = 2 for THP-1 independent experiments were performed in duplicates).

We have previously established a highly sensitive NF-κB::eGFP reporter cell line based on Jurkat JE6-1 cells (39). We examined the activation of these reporter cells by different TLR ligands and found that the reporter cells selectively and strongly reacted with the TLR5 ligand flagellin whereas ligands of other TLRs did not induce significant activation of these reporter cells (**Figure 1A**). Reporter gene expression was concentration-dependent and less than 100 pg/mL flagellin was sufficient to induce detectable eGFP upreglation (**Figure 1B**). Comparison with a monocytic human THP-1-NF-κB::eGFP reporter cell line, established in our laboratory (41), revealed that the Jurkat-based reporter cells were at least as sensitive towards flagellin (**Figure 1C**). These results indicate that in Jurkat JE6-1 reporter cells TLR signaling pathways towards NF-κB activation are operative and furthermore that these cells do not harbor functional cell surface TLRs except TLR5.

### Generation of TLR2/1, TLR2/6 and TLR4/MD-2/CD14 Jurkat NF-κB::eGFP Reporter Cells

**Figure 2:**
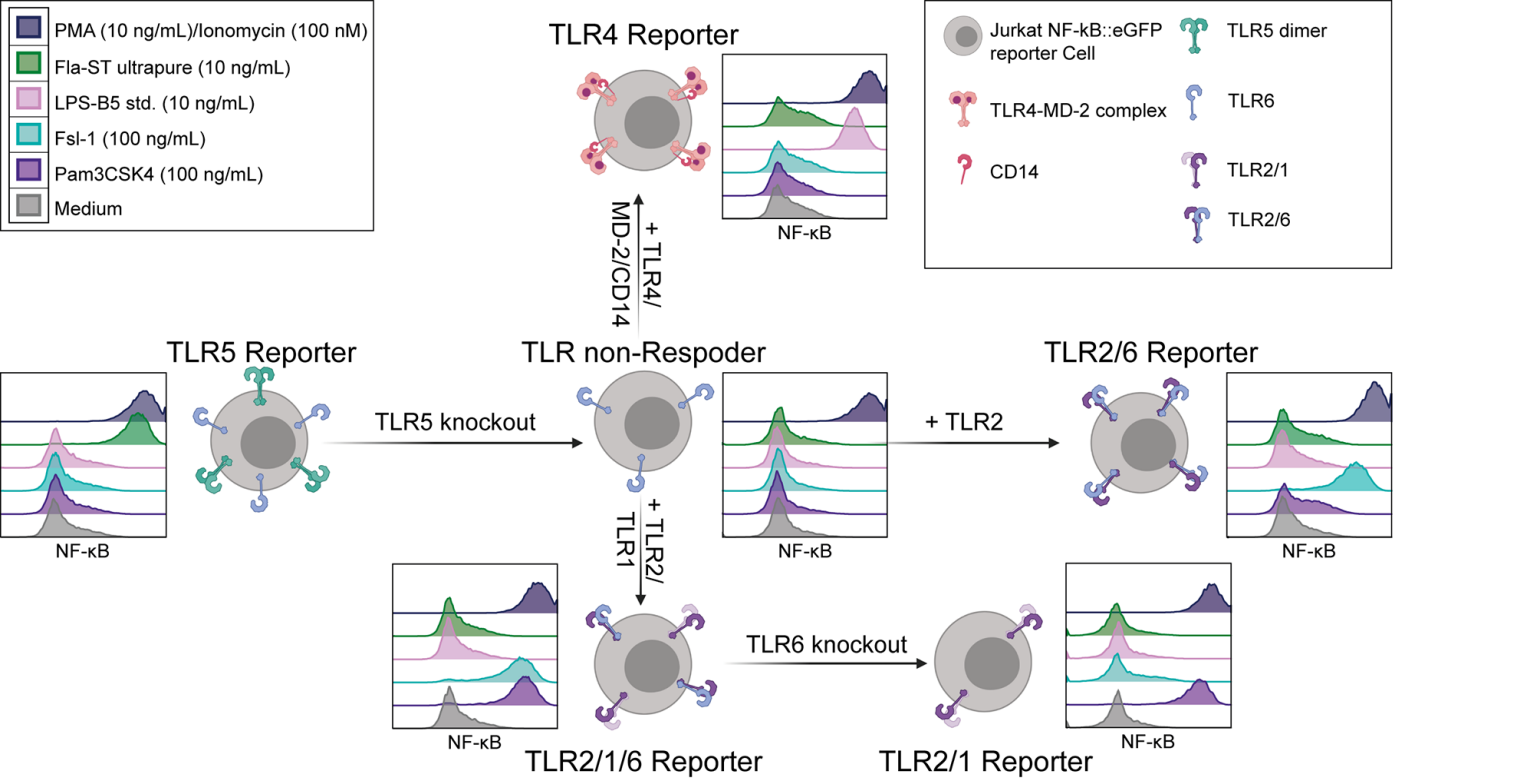
Generation of TLR reporter cells. Scheme for the generation of JE6-1-TLR reporter cell lines (created with BioRender.com) and exemplary histograms representative of at least three independent experiments showing the NF-κB::eGFP expression (standard logarithmic scale) after 24 h incubation with the typical TLR ligands Pam3CSK4 (violet, TLR2/1), Fsl-1 (light blue, TLR2/6), LPS (pink, TLR4) and flagellin (green, TLR5), as well as medium (grey, negative control) and PMA/Ionomycin (dark violet, positive control).

The high sensitivity of the Jurkat reporter cells towards flagellin makes them a promising resource for establishing a set of reporter cells specifically reacting to ligands for different TLR complexes. In a first step we knocked out the endogenous TLR5 gene by CRISPR/Cas9. A clone that was not activated by flagellin but strongly responded to stimulation with PMA/Ionomycin was selected for further use and termed TLR non-responder (**Figure 2**). Retroviral expression constructs encoding cell surface resident TLRs and coreceptors were used to generate highly sensitive and specific TLR reporter from these cells. To generate TLR4 reporter cells, TLR4 and the coreceptors CD14 and MD-2 were introduced into the TLR non-responder reporter cells and single cell clones were established. Extensive screening for cells that were characterized by low background activation and a strong responsiveness towards LPS yielded a highly sensitive TLR4 reporter clone (**Figure 2**). The retroviral transduction of TLR2 into the TLR non-responder reporter cells was sufficient to establish strong reactivity towards the TLR2/6 ligand Fsl-1, indicating endogenous TLR6 expression in Jurkat cells. To generate TLR2-only reporter cells, endogenous TLR6 expression was knocked out by CRISPR/Cas9 in the TLR2/6 reporter cells. Introduction of TLR2 and TLR1 into the TLR non-responder cells generated TLR2/1/6 reporter cells, which reacted to the TLR2/1 ligand Pam3CSK4 but also to the TLR2/6 ligand Fsl-1. A clone with high sensitivity for both ligands was selected for further use. The endogenous TLR6 gene was knocked out in this clone by CRISPR/Cas9 to generate TLR2/1 reporter cells, which did not react with Fsl-1, but were highly responsive towards Pam3CSK4 (**Figure 2**).

### Jurkat TLR Reporter Cells Are Highly Sensitive and Specific

**Figure 3:**
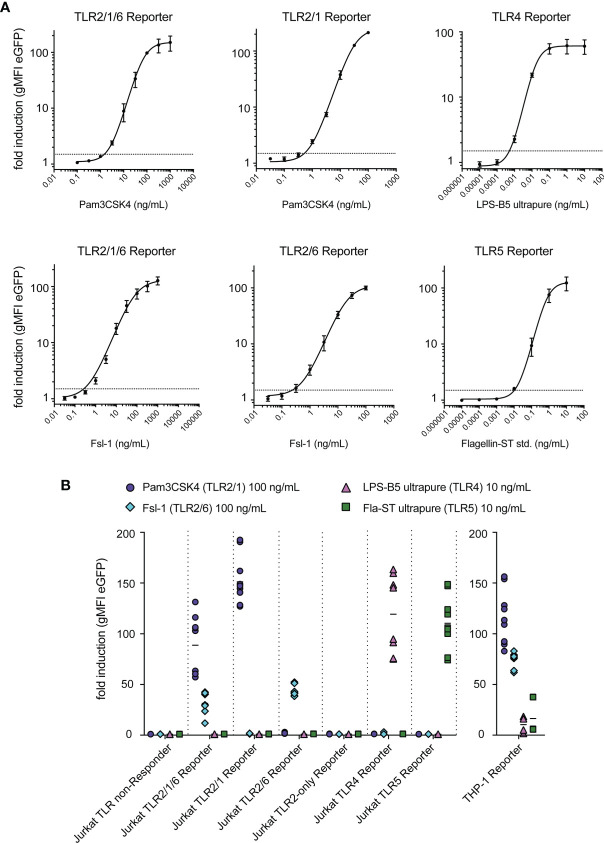
Sensitivity and specificity of TLR reporter cells. **(A)** Titration curves for typical ligands of each TLR reporter cell, with a dashed line at fold induction of 1.5 representing the limit of detection; summary of representative experiments performed in duplicates (n = 2 for TLR2/1/6, TLR2/1, TLR2/6, TLR4 reporter, n = 3 for TLR5 reporter). **(B)** Reactivity of each reporter cell line to the typical ligands of TLR2/1 (Pam3CSK4, 100 ng/mL), TLR2/6 (Fsl-1, 100 ng/mL), TLR4 (LPS-B5 ultrapure, 10 ng/mL) and TLR5 (Fla-ST ultrapure, 10 ng/mL) (n = 6, each experiment was performed in duplicates).

In a next set of experiments, we characterized our JE6-1-TLR reporter cells regarding concentration response, sensitivity and specificity. Each reporter line was stimulated with titrated amounts of the respective ligands. Plotting the geometric mean fluorescence intensity (gMFI) of eGFP expression against the ligand concentration yielded sigmoidal concentration-response curves for each reporter line (**Figure 3A**). Concentrations that induced a 1.5-fold increase of eGFP expression in the reporter cells were estimated to be the limit of detection for each ligand. Based on this assumption the limit of detection of the respective reporter cells was around 1.0 ng/ml for Pam3CSK4; 0.2 ng/ml for Fsl-1; 500 fg/ml for LPS and 10 pg/ml for flagellin (**Figure 3A**).

To confirm their specificity, the JE6-1-TLR reporter lines were stimulated with the typical ligands for TLR2/1 (Pam3CSK4), TLR2/6 (Fsl-1), TLR4 (LPS-B5) and TLR5 (flagellin Fla-ST) at concentrations that elicit a high activation if the corresponding TLR is expressed. We observed that the Jurkat-based NF-κB::eGFP-TLR reporter cells were only activated by their respective ligands. By contrast, NF-κB::eGFP reporter cells based on the human monocytic cell line THP-1, which endogenously express a variety of TLRs (41), were stimulated by all ligands tested (**Figure 3B**).

### Comparison of Different Systems for LPS Detection

**Figure 4:**
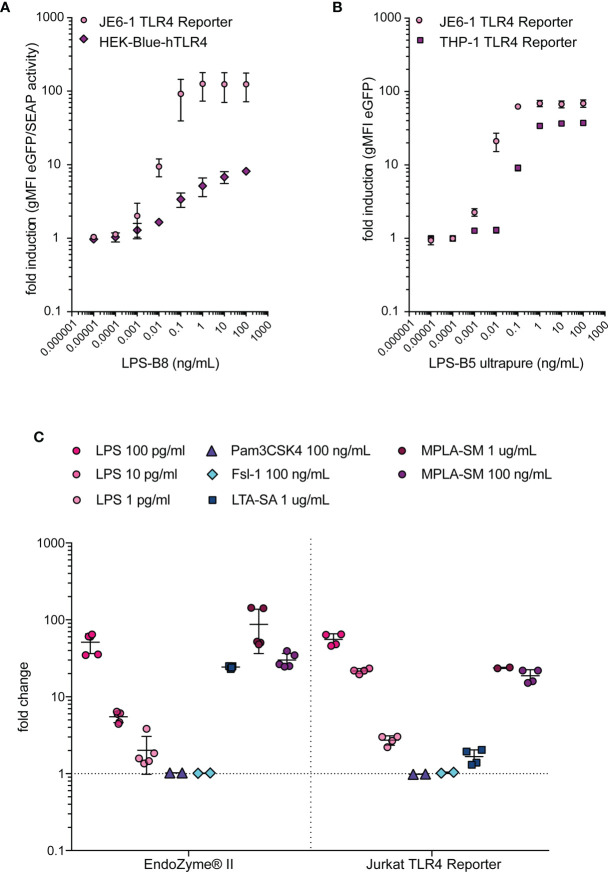
Comparison of LPS-detection systems. **(A)** Fold induction of eGFP expression in JE6-1-NF-κB::eGFP TLR4 reporter cells compared to fold induction of SEAP activity in HEK-Blue™-hTLR4 cells after incubation with different LPS concentrations overnight. Mean and standard deviation of two experiments performed in duplicates are shown. **(B)** Fold induction of eGFP expression in JE6-1-NF-κB::eGFP TLR4 reporter cells and THP-1-NF-κB::eGFP TLR4 reporter cells after 24 h incubation with different concentrations of LPS. Mean and standard deviation of two experiments (JE6-1) or one representative experiment (THP-1) performed in duplicates are shown. **(C)** Reactivity of the typical TLR ligands LPS (LPS-B5 ultrapure, TLR4), Pam3CSK4 (TLR2/1), Fsl-1 (TLR2/6), LTA-SA (TLR2) and MPLA-SM (TLR4) in the recombinant Factor C assay EndoZyme^®^II. The fold change of RFU in the recombinant Factor C assay was compared with the fold induction of gMFI (eGFP) in Jurkat-NF-κB::eGFP TLR4 reporter cells after 24 h incubation with the same ligands (n = 2 independent experiments performed in duplicates or triplicates).

The detection of LPS in biological samples and pharmaceutical products is of utmost importance and, consequently, a wide range of detection systems for this TLR ligand has been developed. We compared the Jurkat TLR4 reporter cells with THP-1-NF-κB::eGFP-TLR4-CD14, which are THP-1 reporter cells engineered to express higher levels of TLR4 and CD14 (41), and the commercially available HEK-Blue™-hTLR4 cells regarding their sensitivity towards LPS. Jurkat TLR4 reporter cells and HEK-Blue™-hTLR4 cells had similar sensitivities towards LPS, whereas the responsiveness of THP-1-NF-κB::eGFP-TLR4-CD14 cells was lower (**Figures 4A, B**). The sensitivity of the Jurkat TLR4 reporter cells was also similar to a standard LPS detection assay based on recombinant Factor C. The recombinant Factor C assay EndoZyme^®^II and Jurkat TLR4 reporter cells do not detect Pam3CSK4 or Fsl-1, the prototypic ligands for TLR2/1 or TLR2/6, respectively (**Figure 4C**). Interestingly, lipoteichoic acid from *S. aureus* (LTA-SA) induces a strong signal in the recombinant Factor C assay and a low response by the JE6-1-TLR4 reporter cells (**Figure 4C**).

### Reactivity of JE6-1 TLR Reporter Cells With Bacterial Cell Wall Components

**Figure 5:**
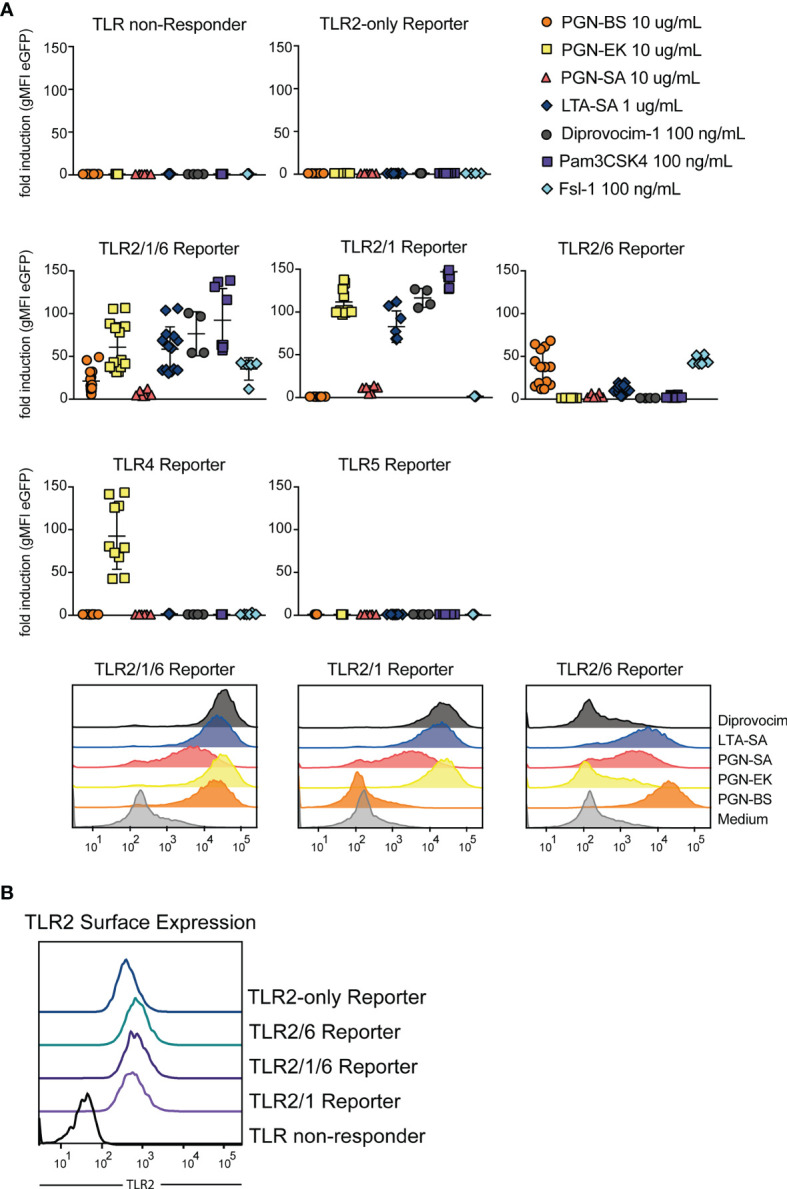
TLR2 stimulatory capacity of different cell wall components. **(A)** Activation of indicated reporter cells by the microbial components peptidoglycan (PGN), isolated from the gram-positive *B. subtilis* (PGN-BS) or *S. aureus* (PGN-SA) or the gram-negative *E. coli* K12 (PGN-EK), purified lipoteichoic acid from *S. aureus* (LTA-SA) and the synthetic ligands Diprovocim-1 (TLR2/1), Pam3CSK4 (TLR2/1) and Fsl-1 (TLR2/6) (n = 8, each experiment performed in duplicates). Exemplary histograms of eGFP expression are shown for TLR2/1/6, TLR2/1 and TLR2/6 reporter cells. **(B)** TLR2 surface expression on different TLR reporter cell lines was compared by staining with an AF647-coupled anti-TLR2 antibody.

We probed the JE6-1-TLR reporter cells with lipoteichoic acid from *S. aureus* (LTA-SA) and peptidoglycans derived from *E. coli* K12 (PGN-EK), *B. subtilis* (PGN-BS) and *S. aureus* (PGN-SA), as well as the synthetic ligands Pam3CSK4, Fsl-1 and Diprovocim-1 (**Figure 5A**). TLR2/1/6 reporter cells were strongly activated by all compounds, whereas TLR2/1 and TLR2/6 differentiated between the PGN-EK and PGN-BS. PGN-EK activated the TLR2/1 reporter cells but not reporter cells expressing the TLR2/6 heterodimers, whereas PGN-BS only stimulated TLR2/6 reporter cells. PGN-SA activated TLR2/1 as well as TLR2/6 reporter cells, but to a lesser degree than PGN-BS or -EK. The PGN-EK preparation also activated TLR4 reporter cells, most likely due to LPS contamination. As Pam3CSK4, Diprovocim-1 was specific for TLR2/1 and TLR2/1/6 reporter cells, but did not activate TLR2/6 reporter cells, whereas Fsl-1 is a ligand specific for TLR2/6. We also generated TLR2-only reporter cells expressing TLR2 without its dimerization partners TLR1 or TLR6 by knocking out TLR6 in the TLR2/6 reporter cell line by CRISPR/Cas9. The TLR2 surface expression was similar to the surface expression of the other TLR2 reporter cells indicating that TLR2 homodimers can reside on the cell surface (**Figure 5B**). Nevertheless, TLR2-only reporter cells were not significantly activated by any of the tested agonists indicating that TLR2 heterodimers are required for the recognition of these compounds (**Figure 5A**).

### JE6-1-TLR Reporter Cells Can Be Used to Detect Contaminations by TLR Ligands

**Figure 6:**
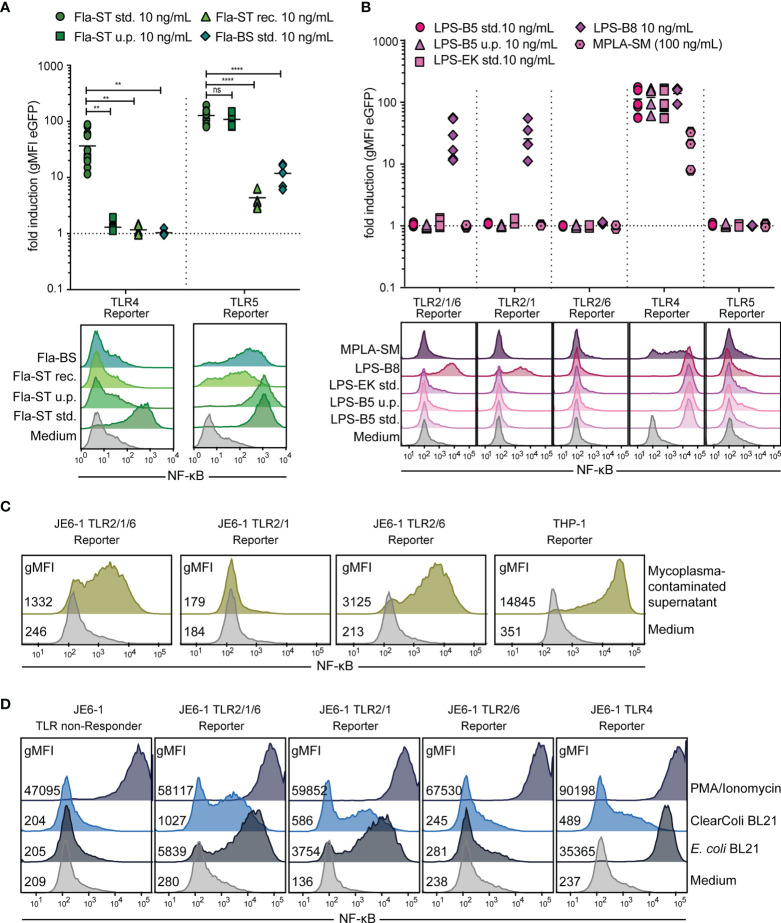
Detection of bacterial contamination. **(A)** Flagellin (Fla) preparations of different purity (std. – standard, 10% purity; u.p. – ultrapure, > 95% purity; rec. – recombinant) from *S. typhimurium* (-ST) or *B. subtilis* (-BS) at 10 ng/mL were compared regarding their capacity to activate TLR5 as well as TLR4 reporter cells. n = 7 independent experiments performed in duplicates and exemplary histograms. ns = p > 0.1; ** = p < 0.01; **** = p < 0.0001. **(B)** Activation of different TLR reporter cells by *E. coli* LPS extracts (10 ng/mL) of different purity (std. – standard, 10% purity; u.p. – ultrapure, > 95% purity) from *E. coli* 055:B5 (-B5), *E. coli* K12 (-EK) or *E. coli* 0127:B8 (-B8), as well as MPLA-SM (100 ng/mL), an LPS component of *S. minnesota*. n = 6 independent experiments performed in duplicates and exemplary histograms. **(C)** Exemplary histograms of eGFP expression in Jurkat-TLR2/1/6, -TLR2/1 and -TLR2/6 reporter cells and THP-1-NF-κB::eGFP reporter cells after overnight incubation with medium or cell culture supernatant containing mycoplasma (1:1 diluted). **(D)** Exemplary histograms of eGFP expression in different TLR reporter cells after overnight incubation with protein preparations of the C4d protein, either expressed in *E. coli* BL21 or ClearColi™ (10 μg/mL) or PMA(100 ng/mL)/Ionomycin(100 nM) as positive control.

We tested different flagellin preparations for their capacity to stimulate our JE6-1-TLR5 reporter cells and for contaminating LPS. Fla-ST standard (std.) and Fla-ST ultrapure are both purified from the gram-negative *S. typhimurium*, with estimated purities of 10% and > 95%, respectively. Both had a high and similar capacity to stimulate TLR5 reporter cells, whereas the reactivity of TLR4 reporter cells towards Fla-ST ultrapure was dramatically reduced as compared to Fla-ST std. (**Figure 6A**). Recombinant Fla-ST expressed in CHO cells and purified by affinity chromatography did not significantly activate JE6-1-TLR4 reporter cells, whereas JE6-1-TLR5 reporter cells were activated, albeit at lower levels compared to Fla-ST std. and ultrapure. As expected, flagellin purified from the gram-positive *B. subtilis* (Fla-BS), does not activate TLR4 reporter cells. Interestingly, this preparation was significantly less potent in activating TLR5 than Fla-ST (**Figure 6A**).

LPS purified by standard methods often contains additional cell wall components that trigger TLR2. We therefore compared different LPS preparations from two different suppliers for their capacity to stimulate TLR4 and TLR2 reporter cells (**Figure 6B**). LPS-B5 std. and LPS-EK are extracted by a phenol-water mixture from *E. coli* O55:B5 or *E. coli* K12, respectively, and are supposed to contain TLR2-stimulatory lipoproteins, as described by the manufacturer. In LPS-B5 ultrapure, lipoproteins are removed by enzymatic hydrolysis. LPS-B8 is purified by gel filtration chromatography from *E. coli* O127:B8 without enzymatic hydrolysis of lipoproteins. Unexpectedly, only LPS-B8, but neither LPS-B5 nor LPS-EK stimulate TLR2/1/6 reporter cells at 10 ng/mL. The stimulation is mediated by TLR2/1 heterodimers, as TLR2/6 reporter cells are not activated by LPS-B8. MPLA-SM, which is extracted from LPS from *S. minnesota* R595 stimulates TLR4 reporter cells, whereas it does not activate TLR2 reporter cells. Compared to LPS-B5, the sensitivity of the JE6-1-TLR4 reporter for MPLA was approximately three orders of magnitude lower (**Figure 6B** and **Figure S1**).

As TLR reporter cells are activated by bacterial components, they can be used to detect bacterial contamination in cell culture, for example by mycoplasma infections. We have previously demonstrated that THP-1-NF-κB::eGFP reporter cells are excellently suited to detect mycoplasma contaminations in cell cultures (41). Mycoplasma contain lipoproteins and lipopeptides, which act as *bona fide* TLR2/6 agonists (46), and we observed that mycoplasma-containing cell culture supernatants activated JE6-1-TLR2/1/6 and -TLR2/6 reporter cells but not JE6-1-TLR2/1 reporter cells. The strongest response was observed with THP-1-NF-κB::eGFP reporter cells corroborating the high utility of this cell line for the detection of mycoplasma contaminations (**Figure 6C**).

LPS is a common contaminant of protein preparations especially when *E. coli*-based expression systems are used. Therefore, *E. coli* strains with genetically engineered LPS have been introduced. One such strain, ClearColi™ BL21, expresses a modified LPS (Lipid IV_A_) that has strongly reduced endotoxicity in eukaryotic cells (47, 48). Human complement split product C4d expressed in standard *E. coli* BL21 cells or *E. coli* ClearColi™ BL21 cells was purified *via* its C-terminal 6xHIS tag, and different JE6-1-TLR reporter cells were probed with the resultant proteins. Jurkat TLR4 reporter cells were barely activated by a protein sample purified from ClearColi™ as compared to the same protein purified from *E. coli* BL21. Nevertheless, the protein purified from ClearColi™ was not devoid of TLR ligands, as it still activated TLR2/1/6 and TLR2/1 reporter cells (**Figure 6D**).

### TLR Reporter Cells Are a Useful Tool to Detect TLR Ligands in Allergen Extracts

**Figure 7:**
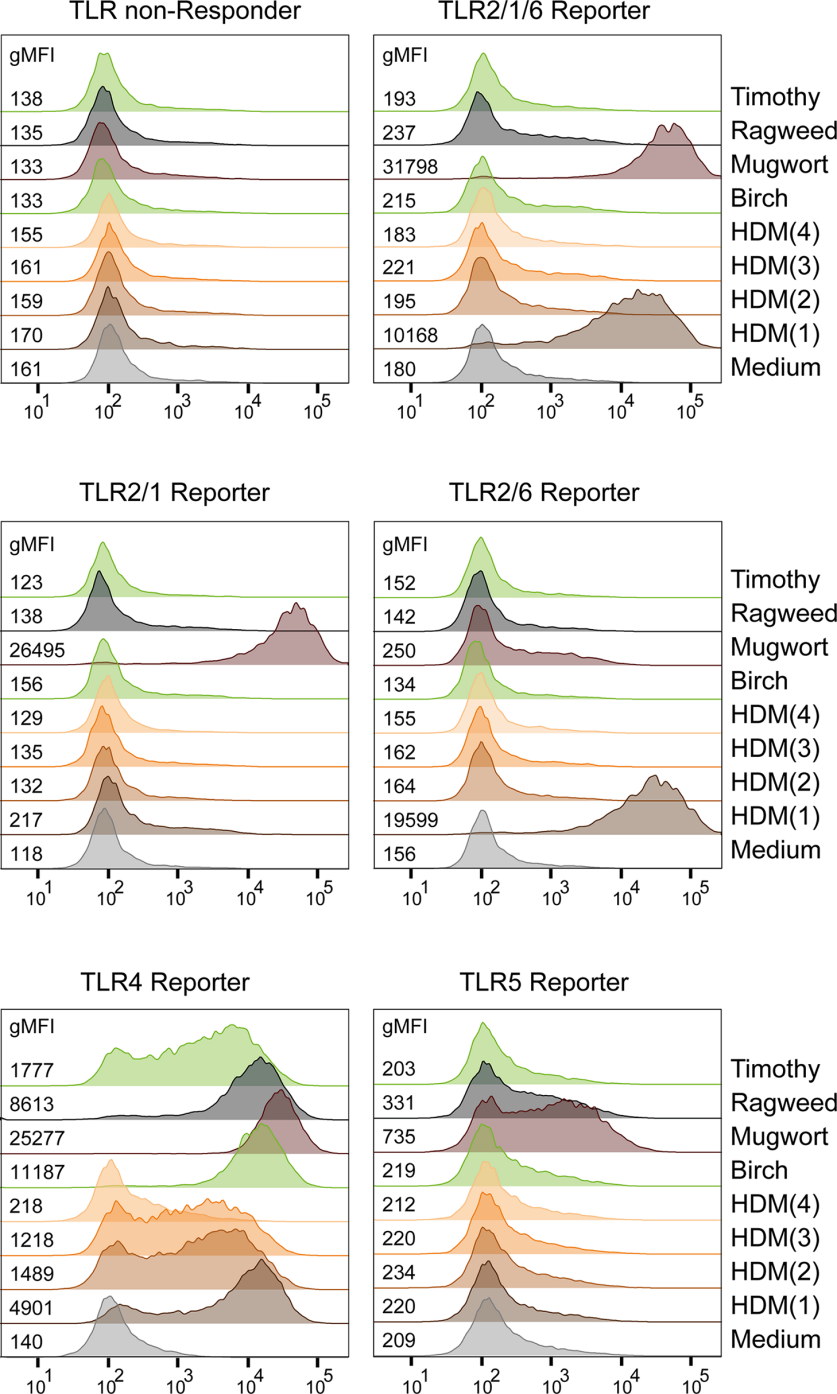
Detection of TLR Ligands in allergen extracts. Exemplary histograms with gMFI of eGFP expression in TLR reporter cells after overnight incubation with allergenic extracts (15 μg/mL) are shown. HDM(1) - HDM(4): house dust mite extracts from different sources.

Although the importance of non-protein constituents contained in allergen sources, such as house dust mite (HDM) or pollen, for allergic responses is well established (49, 50), information regarding the presence of TLR ligands in allergen containing extracts is frequently lacking. We therefore used our set of JE6-1-TLR reporter cells to detect TLR ligands in allergen extracts from different sources. In addition to pollen extracts from timothy grass, ragweed, mugwort and birch pollen we tested four different HDM extracts (*Dermatophagoides pteronyssinus* and *Dermatophagoides farinae*). All extracts contained TLR4 ligands but the amount varied considerably between different extracts. (**Figure 7**). Allergen extracts from mugwort for instance also contained significant amounts of TLR5 and TLR2/1 ligands. When comparing the different HDM extracts, only HDM(1) contained TLR2/6 ligands in addition to TLR4 ligands. Significant differences in the TLR ligand content of proteinacous extracts from allergen sources could have a strong impact on their immunostimulatory capacity.

## Discussion

Contamination with TLR ligands is a major concern for the production of proteins for research but also for protein-based therapeutics. This problem is not limited to the use of bacterial host cells since impurities can also be introduced during the manufacturing process. For example, the proposed immunostimulatory capacity of heat shock proteins turned out to derive, at least in part, from TLR ligands contained in the heat shock protein preparations (21, 23). Although contaminations with LPS are a main concern, the presence of other TLR ligands might also distort the outcome of experiments with recombinant proteins (17, 28, 32). Importantly, trace levels of ligands for TLRs and other PRRs, frequently referred to as innate immune response modulating impurities (IIRMIs) can render therapeutic proteins immunogenic, thereby dramatically increasing the risk of anti-drug antibodies (17, 51–55). Haile et al. demonstrated that clinically used IFN-ß-products, which were reported to be more immunogenic, contained trace levels of TLR2 and TLR4 ligands, whereas such impurities were not detected in IFN-ß-therapeutics with low immunogenicity (34).

LAL assays or equivalent tests based on recombinant Factor C, the main component of the LAL coagulation cascade, are regarded as the gold standard for the detection and quantification of endotoxin in biological and pharmaceutical products. However, serious limitations are associated with the use of these test systems. One of them is its frequently observed inability to detect endotoxin due to masking effects also referred to as “low endotoxin recovery”. Importantly, a multitude of factors including buffer formulation, chelating agents but also the presence of albumin can be causative for low endotoxin recovery (56, 57). In addition, although Factor C and TLR4 both react with high sensitivity to LPS, they are unrelated proteins and, consequently, it has been demonstrated that their capacity to react with LPS from different bacterial strains but also with derivatives of LPS such as MPLA or LPS antagonists might differ considerably. The differences between immune cell-based assays, which rely on TLR4, and LAL-based assays have been demonstrated (58, 59). In addition, as pointed out above, most agonists for TLRs other than TLR4 will not be detected by assays based on limulus amebocyte Factor C. The high costs per sample are another limitation of these types of assays, especially for research laboratories.

Here we describe a reporter cell-based assay system for the highly sensitive and selective detection of ligands for human TLR2/1, TLR2/6, TLR4 and TLR5 complexes. The system is replenishable, very cost effective and requires little hands-on time. It is suitable for high throughput screening strategies for TLR agonists or antagonists. Compounds that mediate TLR-independent reporter activation can be identified using the TLR non-responder reporter cells. Whereas slight differences in the cell numbers impair the accuracy of cell-based reporter assays that rely on bioluminescence or color reactions, our assay, which measures fluorescence on a per cell basis, is not influenced by this factor. In addition, flow cytometric measurement allows to assess the condition of the stimulated reporter cells and thus to exclude assay artifacts which can for instance be caused by toxic compounds.

We have used our Jurkat TLR reporter cells to characterize different commercially available TLR ligands as well as more complex samples like bacterially expressed proteins or allergen extracts. We found that flagellin purified from *S. typhimurium* by acid hydrolysis, heating and ultrafiltration (Fla-ST std.) strongly stimulates TLR4 reporter cells, probably due to residual LPS. This TLR4 stimulatory capacity is not seen with flagellin purified from the gram-positive *B. subtilis* (Fla-BS) by the same purification procedure. Interestingly, Fla-BS is a weaker inducer of TLR5 activation than Fla-ST. Additional purification of Fla-ST by affinity chromatography removes most of the LPS content, retaining strong TLR5-stimulatory capacity. Recombinantly produced flagellin was found to have decreased capacity to activate TLR5 reporter cells compared to flagellin purified from *S. typhimurium*.

To overcome the problem of LPS contamination in proteins expressed in *E. coli*, the most widely used host for recombinant protein production, genetically engineered *E. coli* with reduced endotoxicity have been established. ClearColi™ for instance express only the precursor lipid IV_A_, which does not elicit endotoxicity in humans (47, 48). We could show that indeed, proteins expressed in ClearColi™ had dramatically reduced TLR4 stimulatory capacity, as compared to proteins expressed in *E. coli* BL21. However, the TLR2/1 stimulatory capacity was decreased to a lesser extent, pointing towards residual bacterial components other than LPS. Such TLR ligands, which could critically influence the results of research on protein functions, will not be detected by standard LAL assays.

LPS purified from bacteria often associates with lipopeptides, which activate TLR2. Such ligands were absent from highly purified LPS as expected, but we found that standard LPS preparations from different manufacturers greatly varied in their TLR2 ligand content.

Currently, there is no consensus on several aspects of TLR2-based recognition of bacterial compounds. Although it has been shown that Diprovocim-1, a synthetic agonist, can induce homodimerization of recombinant TLR2 proteins, it is not clear, whether TLR2 homodimers are functional receptors for bacterial cell wall components (60). Following CRISPR/Cas9-mediated knockout of the endogenous TLR6 in JE6-1-TLR2/6 reporter cells, we could isolate clones that stained positive for TLR2, indicating that TLR2 homodimers can be expressed on the cell surface. Nevertheless, these reporter cells did not react with any of the bacterial TLR2 agonists tested. Notably, Diprovocim-1 induced activation of reporter cells expressing TLR2/1 heterodimers, whereas reporter cells expressing only TLR2 were not activated. Therefore, it is likely that heterodimeric pairing of this receptor is required for ligand recognition although we cannot completely rule out that homodimeric TLR complexes react with as of yet unknown ligands. The role of peptidoglycan as TLR2 ligand is also not completely clarified. Travassos et al. found that highly purified peptidoglycans isolated from different bacterial species did not trigger TLR2-mediated reporter cell activation and implicated contaminations with lipoteichoic acids and lipoproteins in the TLR2 activation mediated by crude peptidoglycan preparations (61). Moreover, the structural differences of peptidoglycans and classical TLR2 ligands like lipopeptides also argue against a specific interaction (62). We observed activation of reporter cells expressing TLR2 heterodimers by peptidoglycan preparations from different bacteria but since this material was not highly pure and high concentrations were required, contaminations with lipoproteins cannot be ruled out. Interestingly, peptidoglycan preparations from *E. coli* (gram-negative) and *B. subtilis* (gram-positive) selectively activated TLR2/1 and TLR2/6 reporters, respectively. This would be consistent with contaminations with triacyl and diacyl lipopeptides, which are prevalent in gram-negative and gram-positive bacteria, respectively. Peptidoglycan preparations from *S. aureus* activated both, TLR2/1 and TLR2/6 reporter cells, which is in line with reports that describe the presence of both diacylated and triacylated lipoproteins in this gram-positive bacterial strain (63, 64).

Using allergen-containing extracts derived from pollen and HDM, we demonstrated that with our reporter cells ligands for different TLRs can be readily detected in complex biological samples. The amounts of TLR ligands significantly differed between extracts and analysis of several extracts from HDM yielded significant differences, which could potentially impact on their immunostimulatory capacity.

Further work is required to pinpoint the capability of defined bacterial compounds to trigger different TLRs. Here we present a novel TLR reporter platform based on the human T cell line Jurkat JE6-1 and provide data demonstrating its utility to detect ligands for defined TLR complexes with high sensitivity. To the best of our knowledge, the specificity for distinct TLR complexes has not been demonstrated for any other assay system. Consequently, we believe that our TLR reporter platform might be the readout system of choice to further dissect TLR recognition using synthetic compounds as well as fully defined bacterial components.

## Data Availability Statement

The raw data supporting the conclusions of this article will be made available by the authors, without undue reservation.

## Author Contributions

KR performed the majority of experiments, designed the study and wrote the manuscript. CB performed experiments and supervised experimental work. JL and WP developed methodology and supervised experimental work. SG performed experiments. JS and KH-S provided essential reagents. PS supervised experimental work, designed the study and wrote the manuscript. All authors critically revised the study. All authors contributed to the article and approved the submitted version.

## Funding

This study was supported by funds of the Austrian Science Fund (DK W 1248-B, FWF-P32411 and FWF-P33582-B).

## Conflict of Interest

The authors declare that the research was conducted in the absence of any commercial or financial relationships that could be construed as a potential conflict of interest.

## Publisher’s Note

All claims expressed in this article are solely those of the authors and do not necessarily represent those of their affiliated organizations, or those of the publisher, the editors and the reviewers. Any product that may be evaluated in this article, or claim that may be made by its manufacturer, is not guaranteed or endorsed by the publisher.
