# Supplementary figures and images for: A Highly Sensitive Cell-Based TLR Reporter Platform for the Specific Detection of Bacterial TLR Ligands
Source: Front Immunol. 2022 Jan 11;12:817604. doi: 10.3389/fimmu.2021.817604 (PMC8786796; doi:10.3389/fimmu.2021.817604)

# LPS-B5 ultrapure vs. MPLA-SM

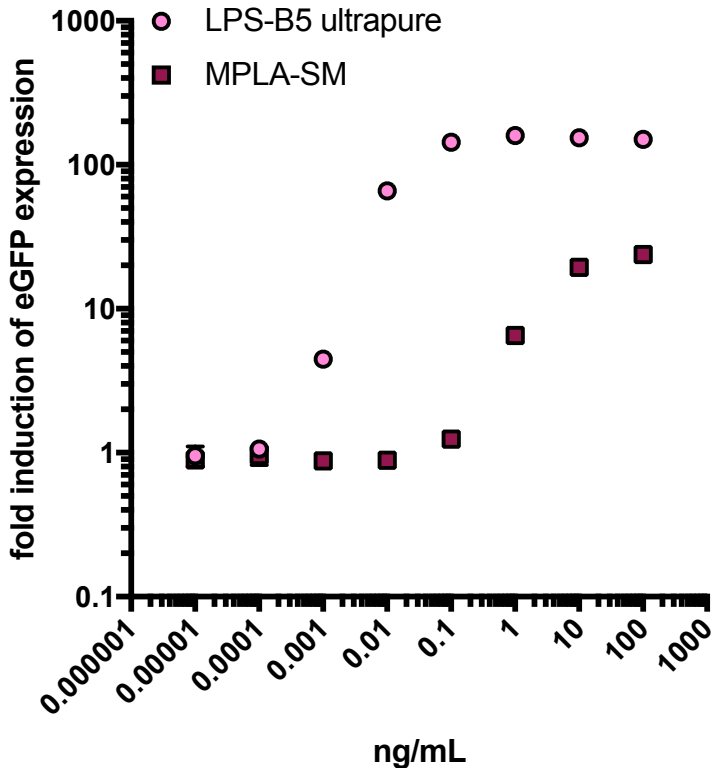

Supplement: Supplementary file 1 [file Image_1.pdf]
