# Supplementary material for: A Highly Sensitive Cell-Based TLR Reporter Platform for the Specific Detection of Bacterial TLR Ligands
Source: Front Immunol. 2022 Jan 11;12:817604. doi: 10.3389/fimmu.2021.817604 (PMC8786796; doi:10.3389/fimmu.2021.817604)
Supplement: Supplementary file 2 [file Table_1.docx]

| Ligand | | Origin | TLR Activation  (according to manufacturer) | Supplier |
| --- | --- | --- | --- | --- |
|  |  |  |  |  |
| CpG | Class B CpG oligonucleotide | synthetic | TLR9 | Invivogen |
| Diprovocim-1 |  | synthetic | TLR2/1 | Sigma Aldrich |
| Fla-BS std. | flagellin standard purification | B. subtilis | TLR5 | Invivogen |
| Fla-ST rec. | flagellin recombinant | S. typhimurium | TLR5 | Invivogen |
| Fla-ST std. | flagellin standard purification | S. typhimurium | TLR5, TLR4 | Invivogen |
| Fla-ST u.p. | flagellin ultrapure | S. typhimurium | TLR5 | Invivogen |
| Fsl-1 | diacylated lipopeptide | synthetic | TLR2/6 | Invivogen |
| Imiquimod (R837) | imidazoquinoline compound | synthetic | TLR7 | Invivogen |
| LPS-B5 std. | lipopolysaccharide standard purification | E. coli 055:B5 | TLR4, TLR2 | Invivogen |
| LPS-B5 u.p. | lipopolysaccharide ultrapure | E. coli 055:B5 | TLR4 | Invivogen |
| LPS-B8 | lipopolysaccharide standard purification | E. coli 0127:B8 | TLR4 | Sigma Aldrich |
| LPS-EK | lipopolysaccharide standard purification | *E. coli* K12 | TLR4 | Invivogen |
| LTA-SA | purified lipoteichoic acid | S. aureus | TLR2 | Invivogen |
| MPLA-SM | monophosphoryl lipid A | S. minnesota R595 | TLR4 | Invivogen |
| Pam3CSK4 | triacylated lipopeptide | synthetic | TLR2/1 | Invivogen |
| PGN-BS | peptidoglycan | B. subtilis | TLR2 | Invivogen |
| PGN-EK | peptidoglycan | E. coli K12 | TLR2 | Invivogen |
| PGN-SA | peptidoglycan | S. aureus | TLR2 | Invivogen |
| Poly(I:C) | dsRNA analog | synthetic | TLR3 | Invivogen |
| Resiquimod (R848) | imidazoquinoline compound | synthetic | TLR7, TLR8 | Invivogen |

**Table S1:** This table shows the TLR ligands that were used in this study, their origin and target TLR.
